# Multimodal Imaging in Iris Vascular Tumors: A Case Series

**DOI:** 10.7759/cureus.31741

**Published:** 2022-11-21

**Authors:** Rocio Eguilior Álvarez, Paula Marticorena-Álvarez

**Affiliations:** 1 Ophthalmology, Hospital Universitario de La Princesa, Madrid, ESP

**Keywords:** multimodal imaging, diagnosis, iris microhemangiomatosis, iris arteriovenous malformation, iris vascular tumor

## Abstract

Iris vascular tumors are very unusual and tend to affect middle-aged and older adults. We report a case series of four adult patients with vascular alterations of the iris. Two patients were diagnosed with simple iris arteriovenous malformation (IAVM) and two with iris microhemangiomatosis (IM). Although the diagnosis is typically clinic, multimodal imaging techniques, especially anterior segment fluorescein angiography (AS-FA), anterior segment optical coherence tomography (AS-OCT), and optical coherence tomography angiography (OCT-A), improve the accuracy and delimitation of their attributes and extension. Differential diagnosis with angle or iris neovascularization, melanoma, and other iris vascular tumors is essential to avoid unnecessary tests and treatments.

## Introduction

Iris vascular tumors are benign lesions whose incidence is low (2% of 3,680 iris tumors described by Shields et al.). According to this study, the most frequent vascular tumors are racemose hemangioma (65%), cavernous hemangioma (12%), varix (9%), microhemangiomatosis (5%), arteriovenous malformation (5%), and pediatric capillary hemangioma (4%) [1]. However, other articles suggest that microhemangiomatosis is more prevalent, due to a large number of clinical cases published [2-4], also taking into account that it is often underdiagnosed.

We present a case series of four adult patients with vascular alterations of the iris and their clinical findings, medical tests, and associated complications.

## Case presentation

This is a case series of four patients (six eyes) under three years of medical follow-up in the ophthalmology department of Hospital Universitario de La Princesa, Madrid, Spain. Patient demographics and referral symptoms were recorded in Table 1.

**Table 1:** Iris vascular tumors: clinical features of the four cases Abbreviations: IAVM, iris arteriovenous malformation; IM, iris microhemangiomatosis; OS, left eye; OD, right eye; OU, both eyes; BCVA, best corrected visual acuity; IOP, intraocular pressure; AMD, age-related macular degeneration

Demographics	Patient 1	Patient 2	Patient 3	Patient 4
Age, years	66	77	46	70
Race	Caucasian	Caucasian	Caucasian	Caucasian
Sex	Male	Female	Male	Female
Referral symptoms	None	None	Decreased vision OD	Decreased vision OD
Tumor type	IAVM	IAVM	IM	IM
Involved eye(s)	OS	OD	OU	OU
BCVA	20/30	20/20	20/30, 20/30	20/40, 20/30
Iris color	Hazel	Green	Brown	Brown
Main iris quadrant	Inferotemporal	Inferotemporal	Diffuse	Diffuse
Clock hours, hours	3-5	7-8	-	-
Episcleral sentinel vessel	0	1	0	0
Number of episodes of microhyphema/hyphema	0/0	0/0	0/1	2/0
IOP, mmHg	16	10	11, 11	13, 14
Associated eye findings	Glaucoma, cataract, scleral buckle	Pseudophakic AMD, dry eye	None	Cataract OU
Associated systemic findings	Hypertension	Immune-mediated necrotizing myopathy	Epilepsy	Type 2 diabetes mellitus

All patients underwent thorough ophthalmologic examination including slit-lamp and dilated funduscopic evaluation, plus gonioscopy. Anterior segment optical coherence tomography (AS-OCT), retinal and anterior segment fluorescein angiography (retinal-FA and AS-FA), and ultrasound biomicroscopy (UBM) were added when necessary. DRI OCT Triton (Topcon, Tokyo, Japan) was used with the anterior segment module to perform AS-OCT and, by removing the module, to perform AS-FA, manually adjusting the focus on the anterior iris. Retinal-FAs were captured using the Carl Zeiss FF450 plus IR Fundus Camera angiographer and the UBM with 50 MHz Aviso S (Quantel Medical, Clermont-Ferrand, France).

Case 1 and 2

**Figure 1:**
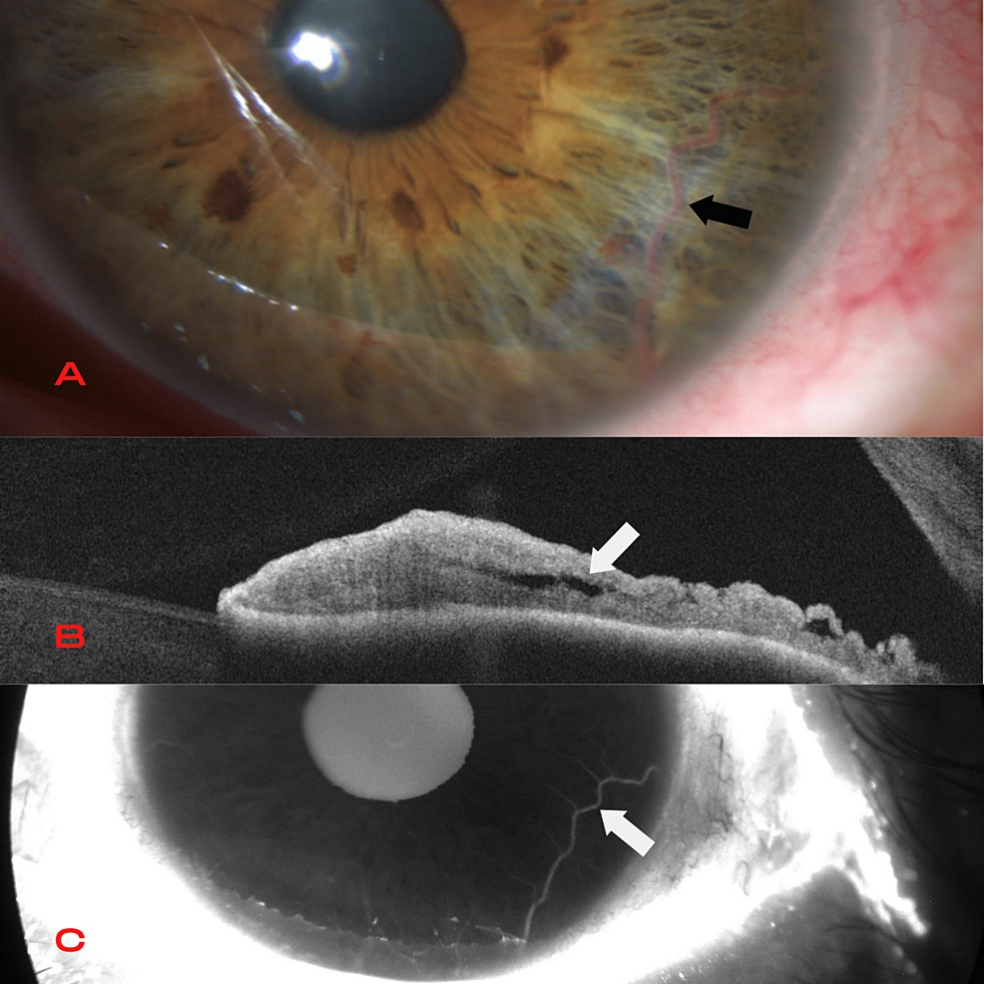
First patient Iris arteriovenous malformation under biomicroscopy (A) as a dilated tortuous iris vessel (black arrow) with a looping course in the iris periphery. Corresponding anterior segment optical coherence tomography (B) shows poorly defined hyporeflective spaces in the iris stroma (white arrow). Early fluorescein angiography (C) exposes a lesion that fills with contrast; it does not present leaking (white arrow).

The first patient, a 66-year-old male, was referred to the anterior segment department due to an iris vascular alteration in his left eye (OS). As medical history, he had high blood pressure controlled with medical treatment. In his OS, he had undergone a scleral buckle and vitrectomy for a retinal detachment three years earlier and was under topical preservative-free latanoprost for high intraocular pressure (IOP). Best corrected visual acuity (BCVA) was 20/30 in his OS. On biomicroscopic examination, the OS showed a thickened and tortuous iris vessel that sprouted from the iris root in the inferior quadrant toward the pupillary margin, running through the stroma and returning to end in the temporal root of the iris (Figure 1A).

**Figure 2:**
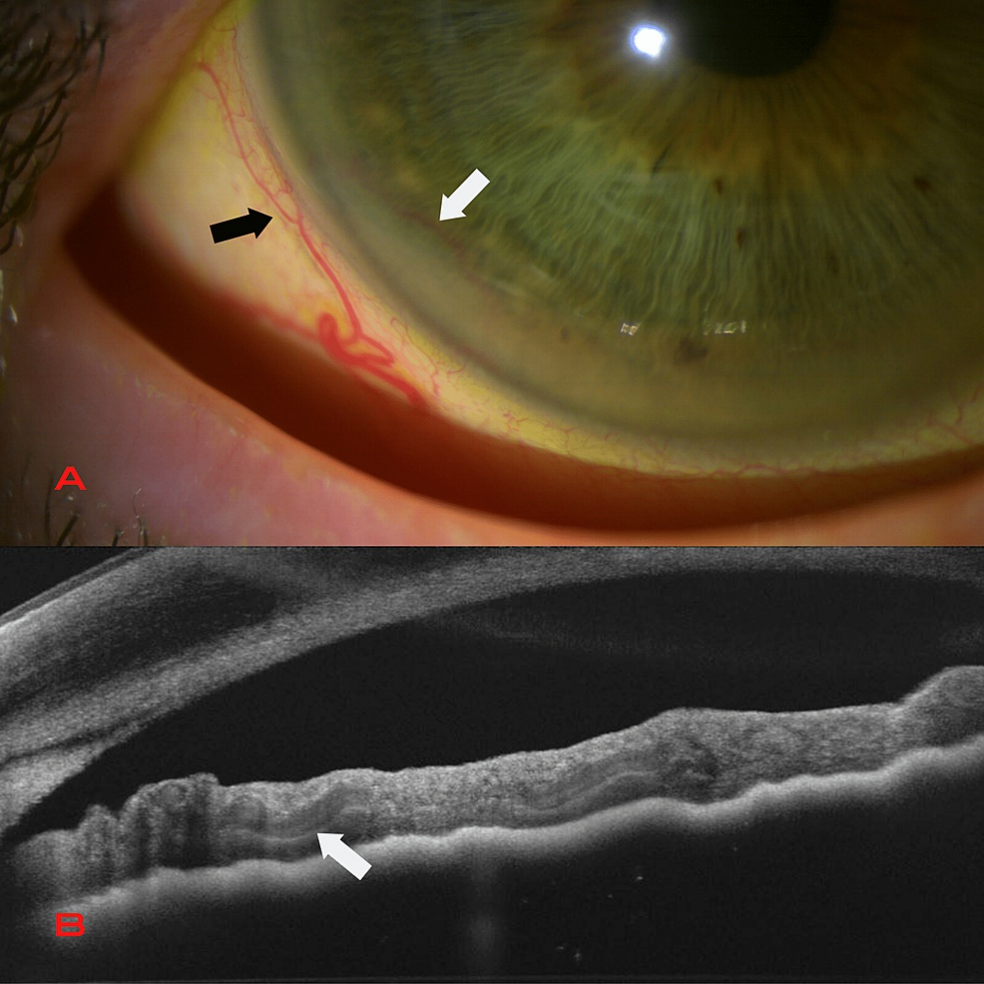
Second patient Iris arteriovenous malformation under slit-lamp photograph (A); there is a dilated tortuous iris vessel (white arrow) with a looping course from the periphery. An episcleral sentinel vessel (black arrow) is seen at that same level of the lesion. Corresponding anterior segment optical coherence tomography (B); the uniform vascular caliber and the serpentine course of the vessel are well visualized in the iris stroma (white arrow).

The second patient, a 77-year-old female, underwent cataract surgery four years earlier with BCVA in both eyes (OU) of 20/20. During a routine visit, an iris vascular lesion was detected in the inferotemporal quadrant of her right eye (OD) with similar characteristics to those described above. An adjacent episcleral sentinel vessel was associated, without continuity between both vessels in gonioscopy (Figure 2A).

AS-OCT allowed visualization of the uniform vascular caliber and the serpentine course of the vessel (Figure 2B), showing poorly defined spaces with low reflectivity in the iris stroma (Figure 1B). AS-FA in both patients revealed a lesion that filled with contrast in the early stages without leaking in the late stages (Figure 1C).

Both patients were diagnosed with simple iris arteriovenous malformations (IAVM). The first patient was asymptomatic during follow-up. The second had an episode of spontaneous subconjunctival hemorrhage in the nasal quadrant of her OD, unrelated to the episcleral vessel.

Case 3 and 4

These patients attended the emergency department reporting non-painful blurred vision in OD. BCVA was 20/30 and 20/40, respectively. A grade one hyphema was detected in the third patient and a microhyphema in the fourth, both spontaneous, with normal eye fundus and IOP. They were treated with topical corticosteroids and cyclopentolate and resolved satisfactorily in one week.

**Figure 3:**
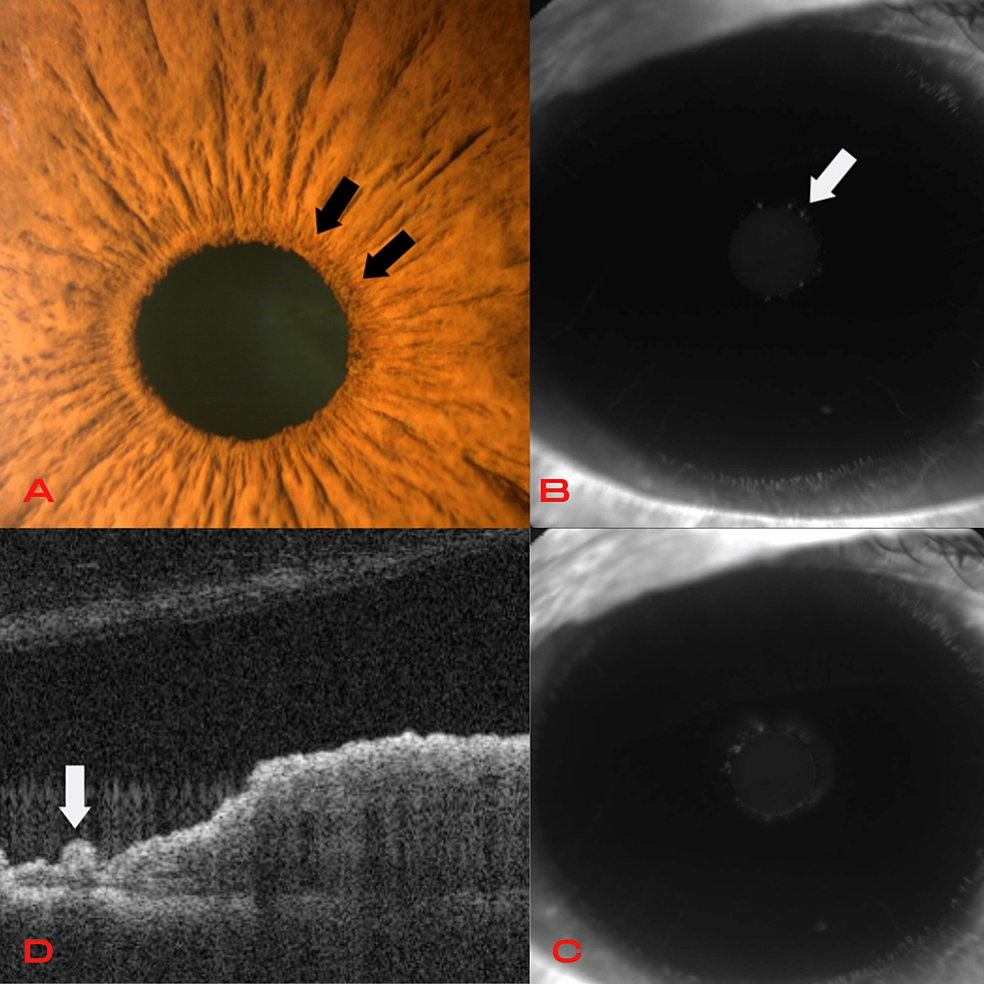
Fourth patient Iris microhemangiomatosis. Slit-lamp examination (A) shows multiple small vascular dilations in the pupillary margin (black arrows) of the right eye and corticonuclear cataract. Fluorescein angiography presents many early hyperfluorescent spots in the pupillary margin (white arrow) (B), with minimal diffusion of dye in the late stage (C). Anterior segment optical coherence tomography (D) locates the lesions in the posterior stroma of the iris (white arrow).

The third patient was an epileptic 46-year-old male, and the fourth was a 70-year-old female with type 2 diabetes without diabetic retinopathy. Slit-lamp examination of these patients showed small vascular dilations in the pupillary margin of OU. AS-FA showed multiple hyperfluorescent spots in the pupillary margin with minimal late diffusion of dye, revealing more lesions than those clinically visible. AS-OCT located the lesions in the posterior stroma of the iris (Figure 3).

Both patients were diagnosed with bilateral iris microhemangiomatosis (IM). The third did not present new episodes of hyphema. The fourth suffered minimal self-limited bleeding in the anterior chamber during OS cataract surgery.

In all four cases, the presence of retinal or angle neovascularization was ruled out by fundoscopy and gonioscopy. Retinal-FA was performed in the second and third patients, as well as retinal OCT angiography (OCT-A) in the third, to rule out retinal neovascularization. In the third and fourth patients, carotid echo-Doppler was done to exclude ocular ischemic syndrome. The second patient, who had the sentinel vessel, underwent a UBM examination to rule out iris or ciliary body tumors.

## Discussion

Iris vascular tumors are benign lesions that tend to arise in middle-aged or elderly adults, usually unilateral, with only 8% of bilateral cases, which are frequently microhemangiomatosis. They are more prevalent in Caucasians without sex predilection [1].

IAVM is a continuity between an artery and a vein without an intervening capillary bed [5,6]. It is more frequently described in light iris, probably because it is easier to detect. Although defined as a congenital lesion, some authors postulate a possible acquired origin, taking into account the average late age of presentation [5]. In our patients, we suspect an acquired origin, since they were adult patients without any lesions in their previous ophthalmologic evaluations.

In biomicroscopy, IAVM appears as a unilateral, single, uniform, and serpentine vessel, with a caliber twice the size of normal iris vessels. It is visible in some areas along the iris stroma, can associate with stromal atrophy, and is usually located in the temporal half of the iris with one or two clock hours of extension [5].

Shields et al. classified IAVM as simple, if the blood vessel forms only one loop, or complex, the most frequent, if overlapping of the vessels is present [5]. No associations have been described with racemose hemangiomas in the retina or brain. However, they report the presence of a dilated episcleral sentinel vessel in 50% of their cases, located in the same quadrant where the iris vascular anomaly is present, without any melanoma or other tumor associated [5,7]. Continuity between the episcleral vessel and the IAVM is suspected, although very few authors have been able to demonstrate it gonioscopically [5].

AS-FA of the IAVM confirms its vascular nature and complete extension. The lesion presents a fast hyperfluorescent filling, without late leakage. Some authors appreciate an adjacent area with little iris vascularization, speculating that IAVM could shunt blood from small nearby capillaries [5]. This test provides the differential diagnosis with neovascularization, which would have a slow leakage of contrast into the aqueous humor [5,7].

AS-OCT in IAVM shows the vessel as a poorly defined hyporeflective structure in the iris stroma [8,9].

OCT-A of the anterior segment distinguishes IAVM from the normal radial vessels of the iris, and it is even superior to AS-FA because its wavelength achieves great penetration into the iris stroma [6]. In UBM, the lesion corresponds to an area of stromal thickening with low reflectivity, which indicates the presence of vascular flow. It is also useful to rule out tumors of the iris or ciliary body [9].

The differential diagnosis of IAVM should include, besides rubeosis iridis, other iris vascular tumors, such as capillary hemangioma, cavernous hemangioma, or varix, which are usually differentiated by their clinical characteristics and systemic associations. Likewise, iris melanoma typically appears as a solid mass with prominent vasculature, so it should always be ruled out in the presence of epibulbar sentinel vessels [5,9].

IAVM usually remains stable and exhibits a benign course, without local complications [5,8].

Otherwise, IM described by Fechner in 1958, also called iris vascular tufts, is a benign idiopathic lesion that occurs at or near the pupillary margin. It consists of unilateral or bilateral hamartomas configured by multiple small vascular dilations that group in clusters of approximately 15-150 μm and present in a multifocal way [2].

It is thought to be an acquired lesion, detected in the sixth and seventh decades of life [3]. The etiology is unknown, but associations have been described with myotonic dystrophy, where they occur at younger ages and in patients with diabetes mellitus, idiopathic juxtafoveal retinal telangiectasia, Sturge-Weber syndrome, hemangioma of the orbit and eyelid, and cerebral aneurysm [3,4,7]. We found an association with diabetes mellitus in the fourth case.

Patients are usually asymptomatic, and most of the time, IM is diagnosed by an episode of blurred vision due to spontaneous bleeding in the anterior chamber that can be associated with increased IOP [3,7].

It is difficult to detect it in biomicroscopy since it is a subtle lesion that requires high magnification. It is believed to be underdiagnosed if it does not cause symptoms. In our case series, two patients presented spontaneous hyphema in one eye, and for this reason, we could detect IM in the asymptomatic contralateral eye.

The diagnostic test of choice for IM is AS-FA. It shows an early slight hyperfluorescence in the pupillary margin with a mildly delayed diffusion of dye, which would represent the ectatic dilatations of the iris vessels. It is often bilateral and without any clear leakage of dye. This test is key for differential diagnosis between IM and neovessels, which would have a clear contrast leak to the anterior chamber and a more diffuse distribution in the iris and would affect the angle. AS-FA also contributes to identifying the real extension of IM, improving the precision of laser treatment, if indicated [2,7].

AS-OCT shows IM located in the posterior stroma of the iris, unlike neovessels, which would be found on the surface [2,4].

The first use of OCT-A in IM was reported by Kang et al. in 2017. This technique shows certain normal-appearing, non-dilated, radial iris vessels, giving rise to tightly coiled vascular tufts at the pupillary margin [4].

Microhemangiomas are large enough to be detected by UBM, but they can be technically difficult, so it is only used in selected cases. They appear acoustically dense with no posterior shadow [2].

The differential diagnosis of IM is similar to the IAVM; besides, in cases of spontaneous hyphema, it should include iris or ciliary body malignant tumors, uveitis-glaucoma-hyphema syndrome, and blood dyscrasias [7].

Both types of iris vascular tumors are typically asymptomatic; hence, observation alone is recommended. One of the most frequent complications described in iris vascular tumors is hyphema. It is more frequent in IM than in IAVM (33% versus 2%). This complication is usually spontaneous and resolves with topical treatment [2,7]. Hyphemas associated with minor trauma, irritation, and cataract surgery have also been reported [2,10]. Our fourth patient presented mild self-limited bleeding during OS cataract surgery, after removing the phacoemulsification tip from the eye, probably triggered by decompression or surgical manipulation. It was solved by increasing the pressure in the anterior chamber with cohesive viscoelastic.

To avoid these hemorrhages during intraocular procedures or postoperative periods, some authors recommend prophylactic photocoagulation of microhemangiomas with argon laser, since hemostasis is achieved faster in a closed eyeball than an open one. AS-FA helps locate better the lesions before applying the treatment [3,10]. Argon laser photocoagulation has been successfully used, not only as prophylaxis but also as a treatment for active bleeding or recurrent hyphemas [3]. Furthermore, treatment with antiangiogenic drugs has been described, but its superiority to observation is not clear [2]. Finally, sectoral iridectomy could be considered when repeated laser treatment fails or in cases where a histopathological diagnosis is crucial to rule out a malignant lesion [8].

## Conclusions

In conclusion, iris vascular tumors are very unusual and tend to affect middle-aged and older adults. Although the diagnosis is typically clinic, multimodal imaging techniques, especially AS-FA, AS-OCT, and OCT-A, improve the accuracy and delimitation of their attributes and extension. Differential diagnosis with angle or iris neovascularization, melanoma, and other iris vascular tumors is essential to avoid unnecessary tests and treatments. IM should be ruled out in all cases of nontraumatic spontaneous hyphema. They are usually asymptomatic and only require observation. However, some may present loss of visual acuity and increased IOP due to spontaneous hyphema, which generally responds well to medical treatment. In cases of incessant active bleeding, recurrent hyphemas, or before cataract surgery, argon laser photocoagulation has been described as an effective treatment.
